
JOURNAL INFORMATION
==============================
NLM Title Abbreviation: Access Microbiol
Journal ID: Access Microbiol
NLM Journal ID: 101746927
Journal ID: acmi

Access Microbiology

EISSN: 2516-8290
Publisher: Microbiology Society

ARTICLE INFORMATION
==============================
PMCID: PMC7481733
Article version: 1
Subjects: Poster Presentation

Abstracts from the Anaerobe Focused Meeting 2019

Publication date: 2019
Electronic publication date: 2019 Sep 30
Volume: 1
Issue: 7
Electronic Location ID: 2
Copyright: © 2019 The Authors
Copyright year: 2019
License: This is an open-access article distributed under the terms of the Creative Commons Attribution License.
License URL: https://creativecommons.org/licenses/by/4.0/

==============================


Investigating Gut Microbiota-Host Interactions in a Microaerobic Environment

http://jmmcr.microbiologyresearch.org

McGrath Conor 1 2 *
Bell Andrew 2
McGrath Emmanuelle 2
Juge Nathalie 2
Schüller Stephanie 1
UEA Medical School , Norwich , United Kingdom
Quadram Institute Bioscience , Norwich , United Kingdom
*Correspondence:Conor McGrath, c.mcgrath@uea.ac.uk

Fastidious anaerobe agar (FAA) as a suitable medium for antimicrobial susceptibility testing (AST) of anaerobes by agar dilution

http://jmmcr.microbiologyresearch.org

Copsey-Mawer Sarah *
Scotford Selina
Copsey-Mawer Bethan
Davis Carol
Perry Michael
Morris Trefor
Hughes Harriet
UK Anaerobe Reference Unit , Public Health Wales , Cardiff
*Correspondence:Sarah Copsey-Mawer, sarahcopsey@hotmail.com

Diversity of the class Coriobacteriia within different ecosystems

http://jmmcr.microbiologyresearch.org

Hoyles Lesley *
Nottingham Trent University , Nottingham , United Kingdom
*Correspondence:Lesley Hoyles, lesley.hoyles@ntu.ac.uk

Engineering a synthetic gut model to explore Clostridioidesdifficile infections

http://jmmcr.microbiologyresearch.org

Hassall Jack *
Unnikrishnan Meera
University of Warwick , Coventry , United Kingdom
*Correspondence:Jack Hassall, j.hassall@warwick.ac.uk

Poo and Puns: The representation of Faecal Microbiota Transplants in English-language print media (2003 – 2017)

http://jmmcr.microbiologyresearch.org

McLeod Carmen *
Nerlich Brigitte
University of Nottingham , Nottingham , United Kingdom
*Correspondence:Carmen McLeod, carmen.mcleod@nottingham.ac.uk

The Sensitivity of PCR combined with the Specificity of Toxin Enzyme Immunoassay: Could an Ultra-sensitive Single Molecule Counting Technology Offer a Standalone Solution for Diagnosis of Clostridioides difficile Infection?

http://jmmcr.microbiologyresearch.org

Perry Michael 1 *
Graham Lee 1
Perry Lauren 1
Parida Sweta 2
Katzenbach Phoebe 1
Baptista Jose 1
Sandlund Johanna 1
Anderson Bethan 1
Copsey Sarah 1
Scotford Selina 1
Morris Tefor 1
Public Health Wales Microbiology , Cardiff , United Kingdom
Cardiff and Vale University Health Board , Cardiff , United Kingdom
Singulex Inc , Alameda , USA
*Correspondence:Michael Perry, michael.perry@wales.nhs.uk

Using PCR in place of GDH as the first line assay in a two-step CDI testing algorithm – evidence of help not hindrance from samples processed at clinical microbiology laboratories in Wales

http://jmmcr.microbiologyresearch.org

Perry Michael *
Anderson Bethan
Perry Selina
Graham Lee
Gilbert Lauren
Birchley Alec
Copsey Sarah
Morris Trefor
Public Health Wales Microbiology , Cardiff , United Kingdom
*Correspondence:Michael Perry, michael.perry@wales.nhs.uk

Evaluation of MICROANAUT-S Anaerobes MIC broth microdilution panels for antibiotic susceptibility testing of anaerobes

http://jmmcr.microbiologyresearch.org

Cordovana Miriam *
Ambretti Simone
University Hospital Policlinico Sant'Orsola-Malpighi , Bologna , Italy
*Correspondence:Miriam Cordovana, miri-78@live.it

Improving the surveillance of antimicrobial resistance trends amongst anaerobic oral pathogens

http://jmmcr.microbiologyresearch.org

Thomas Dafydd 1 *
Scotford Selina 2
Thomas James 3
Crossland James 3
Kelly Grace 4
Morris Trefor 4
Lewis Michael 4
Wilson Melanie 4
Oral Pathology , University Dental Hospital , Cardiff & Vale University Health Board
UK Anaerobe Reference Unit , Public Health Wales , Cardiff
Community Dental Service , Cardiff & Vale University Health Board , Cardiff
School of Dentistry , Cardiff University , Cardiff
*Correspondence:Dafydd Thomas, thomaso9@cardiff.ac.uk

Discovering a novel resistance gene for carbapenem resistance in Parabacteroides timonensis using Whole Genome Sequencing

http://jmmcr.microbiologyresearch.org

Boiten Kathleen *
Veloo Alida
University Medical Center Groningen , Groningen , Netherlands
*Correspondence:Kathleen Boiten, k.e.boiten@umcg.nl

Authenticating Anaerobes – Use of MALDI-TOF MS to identify anaerobes in the NCTC Collection

http://jmmcr.microbiologyresearch.org

McGregor Hannah
Pindoria Dipali
Rai Rupa *
Alexander Sarah
National Collection of Type Cultures , Public Health England , Colindale
*Correspondence:Rupa Rai, rupa.rai@phe.gov.uk

Survival strategies of Clostridium difficile to fluctuating concentrations of oxygen

http://jmmcr.microbiologyresearch.org

Martins Maria Carlos *
Salgueiro Bruno
Carlos Filipe
Romão Célia
Frazão Carlos
Teixeira Miguel
ITQB- Instituto de Tecnologia Química e Biológica António Xavier , Oeiras , Portugal
*Correspondence:Maria Carlos Martins, mariascmartins@hotmail.com

The role of metalloenzymes for the survival of the anaerobe Clostridium difficile during infection

http://jmmcr.microbiologyresearch.org

Folgosa Filipe *
Alves Catarina
Martins Maria C.
Teixeira Miguel
ITQB- Instituto de Tecnologia Química e Biológica António Xavier , Oeiras , Portugal
*Correspondence:Filipe Folgosa, f.folgosa@itqb.unl.pt

